# Deterioration in Global Organization of Structural Brain Networks in Schizophrenia: A Diffusion MRI Tractography Study

**DOI:** 10.3389/fpsyt.2018.00272

**Published:** 2018-06-26

**Authors:** Seung-Hyun Shon, Woon Yoon, Harin Kim, Sung Woo Joo, Yangsik Kim, Jungsun Lee

**Affiliations:** ^1^Department of Psychiatry, Asan Medical Center, University of Ulsan College of Medicine, Seoul, South Korea; ^2^Korea Armed Forces Capital Hospital, Department of Psychiatry, Seongnam, South Korea; ^3^Republic of Korea Marine Corps, Pohang, South Korea; ^4^Graduated School of Medical Science and Engineering, Korea Advanced Institute for Science and Technology, Daejeon, South Korea

**Keywords:** schizophrenia, diffusion MRI, probabilistic tractography, network analysis, connectivity

## Abstract

Schizophrenia is a heterogenous neuropsychiatric disorder with varying degrees of altered connectivity in a wide range of brain areas. Network analysis using graph theory allows researchers to integrate and quantify relationships between widespread changes in a network system. This study examined the organization of brain structural networks by applying diffusion MRI, probabilistic tractography, and network analysis to 48 schizophrenia patients and 24 healthy controls. T1-weighted MR images obtained from all participants were parcellated into 87 regions of interests (ROIs) according to a prior anatomical template and registered to diffusion-weighted images (DWI) of the same subjects. Probabilistic tractography was performed to obtain sets of white matter tracts between any two ROIs and determine the connection probabilities between them. Connectivity matrices were constructed using these estimated connectivity probabilities, and several network properties related to network effectiveness were calculated. Global efficiency, local efficiency, clustering coefficient, and mean connectivity strength were significantly lower in schizophrenia patients (*p* = 0.042, *p* = 0.011, *p* = 0.013, *p* = 0.046). Mean betweenness centrality was significantly higher in schizophrenia (*p* = 0.041). Comparisons of node wise properties showed trends toward differences in several brain regions. Nodal local efficiency was consistently lower in the basal ganglia, frontal, temporal, cingulate, diencephalon, and precuneus regions in the schizophrenia group. Inter-group differences in nodal degree and nodal betweenness centrality varied by region and showed inconsistent results. Robustness was not significantly different between the study groups. Significant positive correlations were found between t-score of color trails test part-1 and local efficiency and mean connectivity strength in the patient group. The findings of this study suggest that schizophrenia results in deterioration of the global network organization of the brain and reduced ability for information processing.

## Introduction

Schizophrenia is a debilitating mental disorder with an onset in early adulthood, a chronic course, and a considerable disease burden (1, 2). Common clinical domains include delusion, hallucination, disorganized thought, and decline of cognitive abilities, with the manifestation and severity of symptoms varying from patient to patient (3). This symptom heterogeneity makes it difficult to understand the pathophysiology of the disease (4). From this background, it is believed that schizophrenia is a disease associated with varying degrees of impairment in a wide range of brain areas, rather than distinct changes in focal brain lesions (5–7). Therefore, for a more objective diagnosis and understanding, a biomarker that can comprehensively analyze schizophrenia-related changes in the whole brain is considered to be important.

Various methods have been used to detect minute changes in brain structure and to analyze them in an integrated manner. Diffusion-weighted imaging (DWI) is a noninvasive method that displays parameters related to the diffusion of water molecules and can be used to provide diffusion tensor image (DTI) by measuring the diffusion of water in many different directions. The diffusion of water in the brain is restricted by cell membranes and cellular structures, and these limitations cause anisotropic diffusion or directional preference in diffusion. Thus, changes in diffusivity measured by DTI can reflect neural tissue damage or the organization of neural fibers. Rather than considering only the single dominant tensor orientation for each voxel, probabilistic tractography (8) repeatedly samples the data and generates many streamline tracts connecting two brain locations, with the density of these tracts reflecting the probability of the voxel-level diffusion directions. However, previous research has revealed that conventional tensor models are subject to several problems with regard to the accurate tracking of complex fiber structures, such as crossing and fanning fibers (9, 10). Tractography methods have therefore been extended to model up to two major diffusion directions in each voxel, with one or two voxel-level directions then being selected for fiber tracking (11). These tractography methods using two-fiber modeling have a higher sensitivity for the detection of multiple fiber populations in the whole brain (11). After generating a set of streamlines for the whole brain, the likelihood of connection between the any two regions can be estimated. By applying tractography, it is possible to reconstruct a brain network that considers the specific regions of the brain as nodes, and the connections between the regions as edges.

Network analysis is a branch of mathematics that describes a system as a graph, which is a set of nodes connected by a set of edges, and then analyses the topological characteristics of the graph (12). The degree of brain structure abnormality between subjects may be quantitatively compared by comparing group differences in metric values obtained from such networks. Network analysis allows researchers to integrate and quantify the relationships in the multivariate data representing the neural network system. Previous studies analyzing reconstructed networks have revealed connectivity disturbances in brain structure in schizophrenia subjects, on the basis of abnormalities in several topological network properties. In short, the main findings concerning the anatomical brain networks of schizophrenia patients were less optimal organization, a less efficiently connected network, less connectivity strength, and reduced hierarchy (13). Researchers have interpreted these results as being suggestive of a decrease in information integration ability and an abnormality in neural development (13–15). Moreover, these findings of altered network organization have led to the hypothesis that structural abnormalities in the brain network could act as biomarkers for inherited genetic vulnerability, and that the more deteriorated the network, the more susceptible it may be to progressive white matter damage, which in turn may bring about more significant functional decline (16, 17).

Robustness is one of the network properties that may be analyzed; it is a concept developed through attempts to understand the brain's stability to physical damage. Robustness simulation, which involves quantifying changes in network properties after removal of components of the brain network, has revealed that the brain is more stable to random damage, but is more vulnerable to target deletion, which is the removal of specific regions in a particular order (18). To our knowledge, less network studies have investigated the robustness of the brains of schizophrenia patients than have examined other network properties, and the results have been inconsistent. Although several previous studies reported that schizophrenia groups showed relatively low resilience to damage compared to healthy control groups (19, 20), there is also one report that found that a healthy control group was affected more severely by the target deletion of nodes (21).

From the viewpoint of the distribution of connectivity, the normal brain network has a topological characteristic in which a relatively small number of regions (i.e., nodes constituting the network) are involved with the majority of connections (22). The “brain hub” (23) is a term that has been used to refer to these specific brain areas, which are deeply involved in the integration of information and have a high degree of centrality. In normal brain network organization, the hub area shows a high degree, a low cluster coefficient, and a high degree of centrality, and is suggested to be an important area contributing to the integration of information and the stability of the network (4, 24). Previous findings have suggested disruption to hub regions, with research strongly implicating a less central position of the prefrontal hubs, and moderately implicating abnormalities of the limbic, temporal, and parietal hubs (4). As the number of hubs in the brain network is relatively small and the importance of each node to the network varies, it is necessary to not only analyze the characteristics of the entire network, but to also compare the characteristics of each of the regional network properties when making comparisons between healthy controls and patients with schizophrenia.

The study of brain networks with diffusion MRI can facilitate the evaluation of the topologic characteristics of the entire brain structure, although it is difficult to accurately reflect the organization of complex brain fibers. Although several network studies have been conducted to date, definitive findings have not yet been achieved. Therefore, to expand the understanding of the structural characteristics of the brain in schizophrenia patients, this study aimed to assess the global network properties of the brain using extended multi-fiber probabilistic tractography. We reconstructed the brain network of each subject using diffusion imaging probabilistic tractography, and then we compared group differences in global network properties. In addition, we compared differences in nodal network properties and conducted a robustness simulation to better understand the stability and regional organization of the brain networks.

## Materials and methods

### Participants

Subjects were recruited from the Asan Medical Center in Seoul, Korea. Forty-eight schizophrenia patients were enrolled, with all patients meeting the Diagnostic and Statistical Manual of Mental Disorders-IV-Text Revision (DSM-IV-TR) criteria. They were all right-handed, between the ages of 20 and 40 years old, and had no other known diseases that could affect brain function. They had all displayed psychotic symptoms such as delusions or hallucinations for <5 years. Twenty-four healthy controls who did not have any Axis I psychiatric diagnosis were enrolled. Furthermore, the healthy controls did not have any first-degree relatives with an Axis I psychiatric diagnosis. In addition, subjects were excluded if they were unable to complete MRI scanning sessions.

Written informed consent was obtained from all subjects. Ethnical approval for the study was obtained from the local Institutional Review Board, Asan Medical Center, Seoul, Korea.

### Neuroimaging acquisition

MRI was performed on a 3-Tesla scanner with an eight channel SENSE head coil (Philips Achieva). Structural T1-weighted images were acquired with a turbo field echo sequence (FOV: 240 × 240 × 170 mm, voxel size: 1 × 1 × 1 mm, TE/TR: 4.6/4.9 ms). DWI images were acquired with an echo planar imaging (EPI) diffusion-weighted sequence. One baseline (b factor = 0 s/mm^2^) image and 32 diffusion-weighted gradient directions (b factor = 1,000 s/mm^2^) were acquired (FOV: 224 × 224 × 135 mm, voxel size: 2 × 2 × 3 mm, TE/TR: 70/5,422 ms, flip angle: 90°). Inappropriate images were found via visual inspection and excluded from further analyses.

Anisotropic voxel affects the distribution of anisotropic signal to noise ratios and can cause directional errors in the fiber tracking algorithm (25). Therefore, DTI images were up-sampled in this study to convert anisotropic voxel size 2 × 2 × 3 mm to isotropic voxel size 2 mm using Slicer V.4.4 (26). Corrections for motion and eddy current artifacts were performed via affine whole brain registration to the b0 baseline using FLIRT in FSL (v 6.0, FMRIB Software, Oxford, UK). During the correction process, gradients or subjects were not discarded by applying a specific threshold. In this study, we did not perform field mapping correction (FDC) such as top up. Since the results of a previous study showed that this approach, which aligns diffusion images to anatomical image as the registration-template without using any additional data such as field-map, provides accurate correction similar to that of the FDC method. (27). The directions of the gradients were compensated for rotations during the process of correction for distortion using affine registration.

T1-weighted images were processed using the Desikan-Killiany atlas of FreeSurfer V 5.3 to parcellate discrete anatomical regions of interest (ROIs) (28, 29). White matter and gray matter ROIs were combined into one ROI for each anatomical structure, resulting in 87 ROIs. This was followed by registration of the parcellated T1-weighted images to the DWI using FLIRT with six degrees of freedom (30).

### Network reconstruction

To track white matter streamlines, we applied a probabilistic tractography method using the Diffusion Toolbox in the FMRIB software library (FSL) (8, 11, 31). Briefly, the probabilistic approach estimates the distribution of the diffusion parameters at each voxel using Metropolis-Hastings Markov chain Monte Carlo (MCMC) sampling (BEDPOST). Then, probabilistic tracking was performed by repeatedly sampling from the distributions. Five thousand samples were performed for each seed ROI and a set of streamlines passing through the given ROI were generated (probtrackx). To reflect the degree of connectivity and the influence of region size, the number of streamline tracts from one seeding ROI that passed through a given second ROI was divided by the total number of generated streamlines (i.e., the way-total value in FSL), to give a connectivity probability for the connection to the second ROI. The non-directional connectivity probability between two ROIs was defined by averaging the two probabilities attained from tracking from each given ROI.

Brain networks were then reconstructed from the collection of ROIs and calculated connectivity probabilities, resulting in an association matrix. Each network was represented as a graph, G = (V, E), consisting of a set of nodes V (representing 87 ROIs) and connections E between the nodes (representing connectivity probability between nodes). To remove weak or spurious connections, we applied a threshold to the connectivity probabilities, with the lowest 10% of connections in each subject's network being discarded. Detailed information on the 87 ROIs used in the study is given in Supplementary Table 1.

### Network examination

Several network properties of the reconstructed brain networks were evaluated to characterize their organization. These network properties were all based on the non-directional weighted matrices and were calculated using the MATLAB-based Brain Connectivity Toolbox (http://www.brain-connectivity-toolbox.net) (32). As the importance of the topological features of each brain region may differ between regions, the nodal network properties were also analyzed.

The nodal local efficiency, degree, and betweenness centrality were calculated to describe the connectivity of the specific brain regions. Nodal local efficiency describes the inverse values of the shortest path length between direct neighbors of a given node, degree represents the number of all connections to a given node, and betweenness centrality is related to the number of shortest paths in a network passing through a given node. Nodal local efficiency quantifies the segregation properties of the network (33), while degree and betweenness centrality allow assessment of the importance of each node (32, 34).

Global properties including the mean connectivity strength, global efficiency, clustering coefficient, local efficiency, and mean betweenness centrality were calculated to define the topological characteristics of the whole brain network. Mean connectivity strength is a global measure of the average connectivity probability values of all connections, and is related to the strength of the entire network (35). Global efficiency is the average of the inverse values of the shortest path length between all pairs of nodes in the network, and quantifies global network integration (33). The clustering coefficient is the global likelihood that the direct neighbors of a given node are interconnected, and quantifies network segregation (36). Local efficiency and mean betweenness centrality were calculated as the average of the node-wise property values.

### Robustness simulation

Robustness is an indicator of network stability when brain damage is present (18). To evaluate the stability of the network, iterative elimination of nodes was simulated. Of the 87 ROIs, 40 nodes were removed one by one in order of decreasing nodal degrees in the brain network of each patient. During node deletion, the global efficiency and global clustering coefficient of each subject's brain network were calculated repeatedly, to track the topological features of the damaged network, after which, group means of changes in network properties according to the number of deleted nodes and differences in group means of differences between patients and control groups were examined. Only 40 ROIs were removed from the network, because the removal of a larger number of ROIs resulted in errors when calculating the clustering coefficient.

### Clinical assessments

Symptom severity was assessed using the Korean version of Positive and Negative Syndrome Scale (PANSS) score (37, 38). To assess cognitive functioning, raw FSIQ scores were estimated using the Korean version of the Wechsler Adult Intelligence Scale (WAIS) and adjusted for age and gender (39). Additionally, color trails test parts 1, 2 (CTT-1,2) were used and the results were presented as T-scores adjusted for age and gender. CTT is an analog of the trails making test (TMT) but is less affected by cultural differences than TMT (40). While CTT part-1 evaluates visuomotor processing speed, CTT part-2 examines attention, visuomotor speed, delayed recall of declarative memory, working memory, and executive functioning (41). Overall psychosocial functioning was assessed using Global Assessment of Functioning (GAF) scale scores (42, 43).

### Group comparison

Demographic data were compared using independent *t*-tests or χ^2^-tests for categorical variables. Differences in network properties between study groups were compared using a two-tailed Student's *t*-test or a Mann Whitney U-test. Multiple comparison corrections were applied to the comparisons of nodal properties by controlling the false discovery rate at 5% (44). A linear mixed model with the number of removed nodes and diagnosis as a fixed effect and subject as a random effect were used to characterize the resilience of the network properties during robustness simulation.

## Results

### Demographic and clinical characteristics

There were no significant differences in mean age or gender between the groups. The mean IQ scores and CTT-2 t scores were lower in patient group than in the healthy control group (*p* < 0.001). Table 1 lists the demographic and clinical characteristics of the participants by group.

**Table 1:** Demographic information on the schizophrenia (SPR) patients and healthy control subjects.

	**SPR**	**Healthy control**	***F* or χ^2^**	***p***
Age, years, mean (*SD*)	28.9 (6.2)	30.0 (5.3)	1.66	0.418
Gender, male, *n* (%)	19 (39.6)	9 (37.5)	0.29	0.864
FSIQ, mean (*SD*)	97.8 (15.5)	120.1 (9.2)	7.86	<0.001
CTT-t 1, mean (*SD*)	48.6(14.4)	54.5(7.5)	1.88	0.064
CTT-t 2, mean (*SD*)	47.0(13.7)	63.8(20.5)	4.15	<0.001
GAF, mean (*SD*)	39.8(19.3)	–	–	–
PANSS, mean (*SD*)	61.0(14.7)	–	–	–

*FSIQ, Full scale intelligence quotient; CTT-t, t-score for Color trails test; GAF, Global assessment of functioning; PANSS, Positive and negative syndrome scale*.

### Global network properties

Patients with schizophrenia showed lower global efficiency, reduced local efficiency, reduced clustering coefficient, and reduced mean connectivity strength in comparison with the healthy control group. Mean betweenness centrality (245.1399 ± 10.2767 vs. 239.6925 ± 10.7912; *F* = 0.013; *p* = 0.041) was higher in schizophrenia patients than in controls. Table 2 lists the group differences in global network properties.

**Table 2:** Global network properties of schizophrenia (SPR) patients and healthy control subjects.

**Network properties**	**SPR**	**Healthy control**	***F***	***p***
	**Mean (*SD*)**	**Mean (*SD*)**		
Global efficiency	1.14E-1 (2.26E-3)	1.15E-1 (2.65E-3)	0.47	0.042
Local efficiency	1.02E-2 (7.87E-4)	1.08E-2 (1.13E-3)	3.22	0.011
Clustering coefficient	7.64E-3 (6.42E-4)	8.12E-3 (9.25E-4)	3.20	0.013
Mean betweenness centrality	245.14 (10.28)	239.69 (10.80)	0.01	0.041
Mean connectivity strength	3.48E-1 (1.28E-3)	3.55E-1 (1.63E-3)	1.97	0.046

### Nodal network properties

None of the findings reached the FDR-threshold. The patient group showed lower nodal local efficiency at an uncorrected (*p* < 0.05) level in several areas, including the basal ganglia (left caudate nucleus and left nucleus accumbens), frontal lobe (left pars orbitalis, right caudal middle frontal gyrus, right medial orbitofrontal cortex, right pars opercularis, right precentral gyrus, and right superior frontal gyrus), temporal lobe (bilateral hippocampus, left superior temporal gyrus, left temporal pole, right inferior temporal gyrus, right superior temporal gyrus, and right transverse temporal gyrus), cingulate cortex (left caudal anterior cingulate cortex, left rostral anterior cingulate cortex, and right rostral anterior cingulate cortex), diencephalon (left thalamus and left ventral diencephalon), and parietal (left precuneus) area.

The schizophrenia group showed increased nodal degree values in the left pars orbitalis, right lateral orbitofrontal cortex, right hippocampus, and right ventral diencephalon regions, while the healthy control group showed increased values in the right transverse temporal gyrus, right supramarginal gyrus, and right nucleus accumbens regions. The nodal betweenness centrality values in the right entorhinal cortex area of the schizophrenia patients were higher than in the healthy control group.

Table 3 lists the ROIs showing differences at uncorrected *p* values of < 0.05 for each of the nodal network properties.

**Table 3:** Regions showing differences in nodal network properties between subject groups (uncorrected level of *p* < 0.05).

**Area**	**ROI name**	**Schizophrenia**		**Health controls**		**Uncorrected *p***
		**mean**	***SD***	**mean**	***SD***	
**NODAL LOCAL EFFICIENCY**						
Frontal	Left pars orbitalis	4.26E-03	7.85E-04	4.90E-03	1.11E-03	0.012^*^
	Right caudal middle frontal gyrus	1.15E-02	1.60E-03	1.24E-02	1.78E-03	0.049^*^
	Right medial orbitofrontal cortex	1.10E-02	1.57E-03	1.23E-02	1.76E-03	0.006^*^
	Right pars opercularis	8.87E-03	1.42E-03	9.86E-03	1.68E-03	0.007^*^
	Right precentral gyrus	1.67E-02	2.61E-03	1.80E-02	2.76E-03	0.032^*^
	Right superior frontal gyrus	2.12E-02	2.27E-03	2.25E-02	2.56E-03	0.022^*^
Temporal	Left hippocampus	9.04E-03	1.66E-03	1.00E-02	2.07E-03	0.220^*^
	Left superior temporal gyrus	1.24E-02	1.44E-03	1.31E-02	1.35E-03	0.039^*^
	Left temporal pole	6.95E-03	1.69E-03	7.99E-03	2.25E-03	0.045^*^
	Right hippocampus	9.95E-03	1.55E-03	1.10E-02	1.72E-03	0.018^*^
	Right inferior temporal gyrus	7.82E-03	1.12E-03	7.99E-03	1.23E-03	0.017^*^
	right superior temporal gyrus	1.16E-02	1.53E-03	1.27E-02	1.50E-03	0.005^*^
	Right transverse temporal gyrus	6.96E-03	1.03E-03	7.60E-03	1.22E-03	0.018^*^
Parietal	Left precuneus	9.74E-03	1.65E-03	1.04E-02	1.25E-03	0.024^*^
Cingulate	Left caudal anterior cingulate cortex	1.19E-02	1.94E-03	1.30E-02	2.06E-03	0.013^*^
	Left rostral anterior cingulate cortex	1.22E-02	1.86E-03	1.33E-02	2.10E-03	0.019^*^
	Right rostral anterior cingulate cortex	1.25E-02	1.88E-03	1.40E-02	2.33E-03	0.008^*^
Basal ganglia	Left caudate nucleus	1.27E-02	1.84E-03	1.41E-02	2.46E-03	0.033^*^
	Left nucleus accumbens	8.37E-03	1.55E-03	9.44E-03	1.82E-03	0.025^*^
Diencephalon	Left thalamus	1.18E-02	1.76E-03	1.26E-02	1.84E-03	0.049^*^
	Left ventral diencephalon	1.05E-02	1.02E-03	1.12E-02	1.47E-03	0.045^*^
**NODAL DEGREE**						
Frontal	Left pars orbitalis	61.10	4.35	58.67	5.74	0.048^**^
	Right lateral orbitofrontal cortex	78.40	3.22	76.58	2.75	0.007^**^
Temporal	Right hippocampus	83.06	2.45	82.79	1.06	0.030^**^
	Right transverse temporal gyrus	77.60	3.47	80.04	2.16	0.003^*^
Parietal	Right supramarginal gyrus	80.85	2.32	82.00	1.67	0.050^*^
Basal ganglia	Right nucleus accumbens	76.98	4.14	79.13	2.88	0.033^*^
Diencephalon	Right ventral diencephalon	84.54	1.03	83.71	1.60	0.027^**^
**BETWEENNESS CENTRALITY**						
Parietal	Right entorhinal cortex	44.83	38.99	23.92	22.13	0.025^**^

*The Mann-Whitney U test was used for all statistical comparisons*.

* *healthy control > schizophrenia*.

** *schizophrenia > healthy control*.

### Robustness of brain structural networks

**Figure 1:**
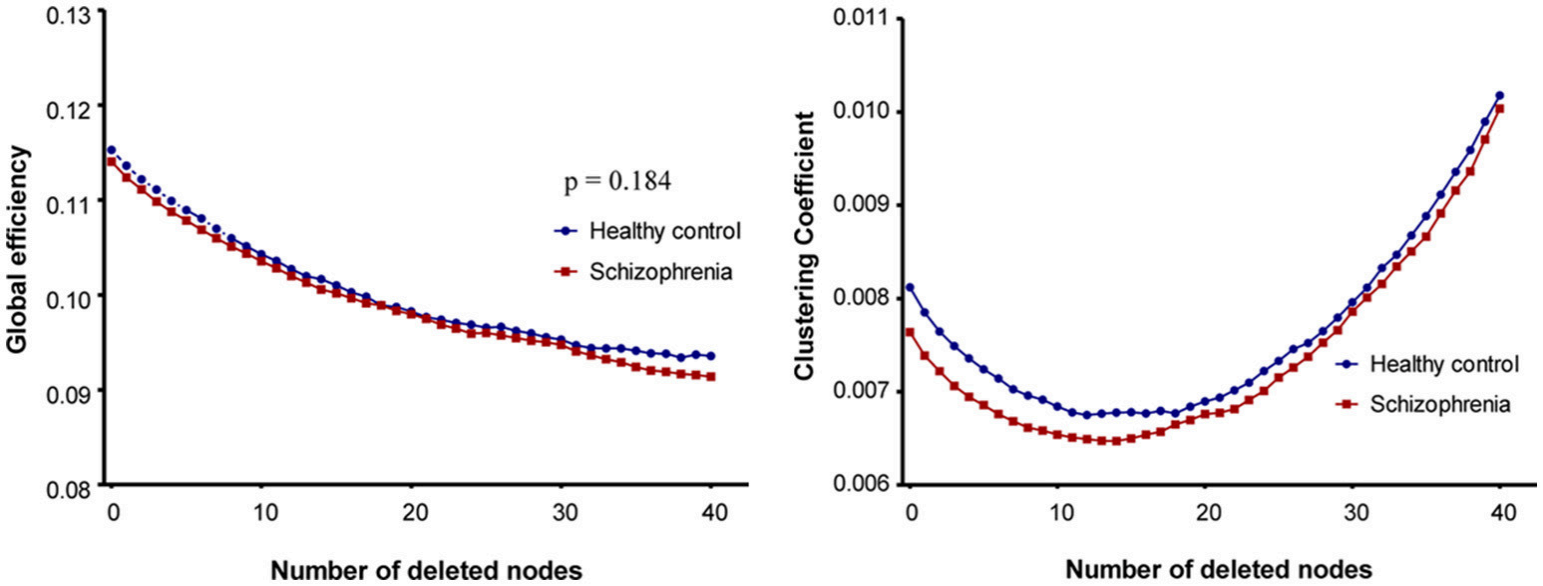
Plots of robustness analysis in schizophrenia patients and healthy control subjects. In case of global efficiency, a linear mixed model was used to assess the group-by-number of removed nodes interaction.

Scatter plots of the number of deleted nodes and changes in network properties illustrate the resilience of the structural brain networks in the two study groups (Figure 1). Global efficiency decreased continuously as the number of removed nodes increased. However, global efficiency did not show a significant group-by-number of deleted node interaction (*p* = 0.184). Plotting of the clustering coefficient resulted in a U-shaped pattern in which the value of the property decreased in the early stage of the simulation, but then increased as the simulation progressed. In the case of the clustering coefficient, robustness could not be compared by linear mixed analysis because group-specific variation of the property did not show linearity.

### Relationship between network characteristics and clinical assessments

In the patient group, there were significant positive correlations between CTT-1 t-score, and local efficiency and mean connectivity strength. The negative correlation of CTT-1 t-score with mean betweenness centrality was significant. CTT-2 t-score had a significant positive association with mean connectivity strength. In the patient group, there were no significant associations between IQ, GAF, and total PANSS score and network properties. In the control group, IQ raw score was significantly positively related to local efficiency, clustering coefficient, and mean connectivity strength.

Table 4 lists the relationship between network characteristics and clinical assessments for each subject group.

**Table 4:** Relationship between network characteristics and clinical assessments.

		**Schizophrenia**		**Healthy Control**	
		***r***	***P***	***r***	***P***
Global efficiency	FSIQ (raw)	−0.09	0.569	0.33	0.138
	FSIQ (adjusted)	0.11	0.457	−0.22	0.327
	CTT-t 1	0.30	0.053	0.41	0.062
	CTT-t 2	0.19	0.259	0.17	0.471
	PANSS	−0.08	0.598		
	GAF	−0.15	0.431		
Local efficiency	FSIQ (raw)	0.16	0.286	0.51	0.016^*^
	FSIQ (adjusted)	0.20	0.174	−0.10	0.642
	CTT-t 1	0.31	0.046^*^	0.40	0.076
	CTT-t 2	0.31	0.056	−0.14	0.539
	PANSS	0.05	0.741		
	GAF	−0.14	0.468		
Clustering coefficient	FSIQ (raw)	0.18	0.238	0.52	0.014^*^
	FSIQ (adjusted)	0.19	0.198	−0.09	0.677
	CTT-t 1	0.30	0.056	0.39	0.081
	CTT-t 2	0.31	0.062	−0.15	0.509
	PANSS	0.07	0.675		
	GAF	−0.14	0.465		
Mean betweenness centrality	FSIQ (raw)	−0.04	0.773	−0.20	0.368
	FSIQ (adjusted)	0.01	0.942	0.32	0.152
	CTT-t 1	−0.34	0.025^*^	−0.23	0.309
	CTT-t 2	−0.21	0.201	0.01	0.951
	PANSS	0.07	0.656		
	GAF	0.15	0.448		
Mean connectivity strength	FSIQ (raw)	0.11	0.471	0.49	0.020^*^
	FSIQ (adjusted)	0.19	0.206	−0.12	0.602
	CTT-t 1	0.43	0.005^*^	0.35	0.120
	CTT-t 2	0.36	0.028^*^	−0.13	0.579
	PANSS	0.03	0.868		
	GAF	−0.13	0.517		

FSIQ, Full scale intelligence quotient; CTT-t, t-score for Color trails test; GAF, Global assessment of functioning; PANSS, Positive and negative syndrome scale

* *p < 0.05*.

## Discussion

The schizophrenia group showed significantly lower values than the control group in global efficiency, local efficiency, clustering coefficient, and mean connectivity strength. Global efficiency is an indicator of network integration, and reduced values could imply that the ability for functional integration across the overall network is degraded. The clustering coefficient and local efficiency value were both lower in the schizophrenia group, which means that the degree of segregation across the overall network, i.e., the local connectedness, was lower. In addition, decreased overall connectivity strength was also reported, suggesting that overall connectivity between regions in the schizophrenia group was different to that in the control group. In a previous study of brain anatomical networks in drug naïve schizophrenia patients, Zhang et al. suggested that decreased connectivity strength in subnetworks affects the deterioration of global topological characteristics (19), which is in accord with our findings.

As suggested in a previous report (24), decreased global network parameters may result from either reduced inter-regional connectivity between brain regions, or disconnections in longer pathways. Our results are mostly consistent with previous studies, although different thresholds and anatomical templates have been applied (45, 46). However, when comparing properties related to network segregation (such as the clustering coefficient) with previous studies, between-study differences can be noted in the schizophrenia patients. (15, 45–47). Differences in tractography, thresholding, imaging sequence, and edge weight may have influenced these between-study differences related to network segregation. Exceptionally, the mean betweenness centrality of this study was significantly higher in the schizophrenia patients than the control subjects. This is inconsistent with previous studies, which showed that schizophrenia may be associated with abnormal network hub organization (4, 48). It has been reported that changes in centrality in schizophrenia are inconsistent between brain regions (24, 45). Thus, the effect on the mean betweenness centrality of schizophrenia might vary from region to region of the brain, and comparisons of the nodal measures should be considered for appropriate comparisons.

In comparison with the normal control subjects, the schizophrenia patients showed significantly lower nodal local efficiency values (at uncorrected *p* < 0.05 levels) in the basal ganglia, frontal, temporal, cingulate, diencephalon, and precuneus regions. Previous studies have implicated these regions in the pathophysiology and symptom manifestation of schizophrenia, with the basal ganglia showing altered integration in schizophrenia, and striatal functional connectivity having been reported as a potential biomarker for predicting response to antipsychotics (49–51). Frontal and temporal areas are reported to show abnormalities in network connectivity in schizophrenia patients, especially in the temporal pole (24). In the thalamus, disruption of cortico-thalamic connectivity is associated with the manifestation of various symptoms of schizophrenia (52, 53). Furthermore, the precuneus, prefrontal, temporal, and anterior cingulate areas are putative network hubs in the brain network, and aberrant hub organization has appeared in previous analyses of brain networks in schizophrenia (4, 13, 48, 54). We suggest that a reduction in the properties related to regional network segregation in schizophrenia may contribute to the alteration of the global network organization, with the extent of the alteration being more prominent in certain areas.

The nodal centrality indicators, as well as the mean betweenness centrality, showed results inconsistent with previous studies. While we found that the nodal degree value in the frontal and hippocampus areas and the betweenness centrality value in entorhinal cortex were rather increased in the schizophrenia group, previous studies found decreased degree in the frontal hub (55), and decreased values of normalized betweenness centrality in some areas related to the default network (15). These discrepancies in the properties related to centrality may be affected by differences in thresholding of the networks and the heterogeneity of the subject groups.

In the case of continuous network damage, a change in a parameter reflects the overall performance, such as the robustness and stability of the network (18). When evaluating robustness using global efficiency, there was no significant interaction between the number of removed ROIs and the study group. To assess the robustness of anatomical brain networks, a previous study used area-under-the-curve analyses with plots based on the largest cluster size and fraction of node deletions, and detected decreased robustness in schizophrenia (19). Differences in the methods for comparing robustness and image processing might have affected the discrepancies in the results between studies. The clustering coefficient showed a change of U shape when the ROI was repeatedly removed, which is suggestive of a limitation in the use of linear mixed models to compare robustness. This limitation is considered to be due to the reduced number of possible connections between neighboring nodes, which is a denominator in calculating the clustering coefficient.

A significant positive association between CTT-1 t-score and local efficiency and mean connectivity strength was found in the patient group, which is partly consistent with previous studies examining the relationship between processing speed measured by the verbal fluency test and the functional brain network (21). In other words, the degree of network segregation and connectivity strength could be an objective measure of visuomotor speed. Mean connectivity strength was positively correlated with CTT-2 t-score, suggesting that connectivity strength may also be related to executive function and working memory. By contrast, mean betweenness centrality was negatively correlated with CTT-1 t-score. IQ raw score in the control group showed a significant correlation with network properties, such as local efficiency, clustering coefficient, and mean connectivity strength, which is consistent with the results of a previous study (47).

However, the network properties showed no significant associations with GAF and PANSS total score or a significant relationship with PANSS positive and negative subscale scores in further analysis. These results are inconsistent with those of previous reports showing associations between symptom severity and reduced levels of overall connectivity and global efficiency (56, 57). Further studies, using both anatomical and functional network analysis in combination with a study of the relationship between baseline network characteristics and longitudinal outcome, will be needed to address this issue more clearly.

Although structural connectivity is closely related to functional connectivity (58, 59) previous network studies showed inconsistency between these two connectivities of brain network (57). In agreement with our study, many previous studies have reported consistently reduced structural connectivity in schizophrenia patient group (59–61). However, studies of functional connectivity have reported decreased connectivity (21, 62), although increased connectivity has also been reported, particularly with respect to hyper-connectivity in the frontal area (63, 64). In addition, although no significant differences were found in the present study, robustness to targeted attack on network hubs in schizophrenia was found to be decreased in anatomical network analysis (19, 45), and increased in functional network analysis (65). These differences cannot be adequately explained by methodological issues alone. Future research, using DTI and fMRI simultaneously and investigating the difference between structural and functional networks, will be needed to expand understanding of how functional connectivity affects structural connectivity.

There are some limitations to this study which need to be addressed. First, as the diffusion imaging technique relies on water diffusion parameters and its spatial resolution is relatively low compared to the actual size of nerve fibers, diffusion MRI has difficulties with resolving complex fiber organizations, such as crossing, converging, diverging, and kissing fibers (66, 67). Nevertheless, diffusion imaging is currently one of only a very small number of tools that can be used for the *in vivo* evaluation of structural networks in the human brain. In addition, to increase the sensitivity of fiber reconstruction, we used a direct extension of a probabilistic tractography method to reconstruct the networks. The extended probabilistic tractography with a crossing fiber model improved sensitivity for capturing the complexity of neural fiber organization (11). Second, most of the participants in the schizophrenia group had previously been administered antipsychotic medications, and the possibility that the medication served as a confounding factor in the presented results cannot be excluded. However, in some previous studies, changes in brain connectivity were also observed in medication naive patients (19). Third, no information was available on the level of cognitive function before the onset of disease in the schizophrenia group, and there was a difference in IQ scores between the study groups at recruitment. Therefore, it is unclear whether the differences in network characteristics between groups were influenced by the progression of the disease or differences in cognitive functions present before the onset of schizophrenia. In future studies, it would be helpful to use neurocognitive tests such as the Wide Range Achievement Tests, which could estimate the premorbid intellectual function of the patient and allow clearer interpretation of the results. Finally, we used a relatively small sample size, which may have resulted in poor reproducibility. Thus, a larger sample size in follow-up studies will be required to demonstrate clear and precise differences in nodal network properties between groups, and to determine whether the conclusions of this study can be generalized to other schizophrenia patients.

## Conclusion

We compared brain structural networks between schizophrenia patients and healthy controls, using diffusion MRI probabilistic tractography and graph theory. When the topological network properties were compared, measures related to the global network integrity and segregation were significantly lower in the schizophrenia group. This suggests that schizophrenia could induce damage to the entire brain structure and deterioration of inter-regional connectivity, which results in less effective network organization. In addition, considering that group differences in nodal local efficiency were prominent in several regions, schizophrenia may be a disease characterized by network damage over a wide range of brain areas, although the damage disproportionally affects specific brain regions.

## Author contributions

S-HS and JL: Conceptualization; S-HS, WY, and HK: Acquisition of data; S-HS, JL, SJ, and YK: Formal analysis; S-HS, JL, WY, HK, SJ and YK: Investigation; S-HS and JL: Original draft; All authors contributed to and approved the final manuscript.

### Conflict of interest statement

The authors declare that the research was conducted in the absence of any commercial or financial relationships that could be construed as a potential conflict of interest.

## Abbreviations

ROIs: Regions of interests
DWI: diffusion-weighted images
DTI: diffusion tensor image
MCMC: Markov chain Monte Carlo.
