# Supplementary material for: Deterioration in Global Organization of Structural Brain Networks in Schizophrenia: A Diffusion MRI Tractography Study
Source: Front Psychiatry. 2018 Jun 26;9:272. doi: 10.3389/fpsyt.2018.00272 (PMC6028716; doi:10.3389/fpsyt.2018.00272)
Supplement: Supplementary file 1 [file Table_1.DOCX]

***Deteriorated Global Organization of Structural Brain Networks in Schizophrenia: A Diffusion MRI Tractography Study***

Seung-Hyun Shon, Woon Yoon, Harin Kim, SungWoo Joo, Yangsik Kim, Jungsun Lee*

*Correspondence: Jungsun Lee: js_lee@amc.seoul.kr

**Supplementary Table 1** List of the 87 Regions of Interest used in the study

All regions except corpus callosum have left/right hemisphere counter-parts

| Region | Area |
| --- | --- |
| Posterior corpus callosum | Corpus callosum |
| Middle posterior corpus callosum | Corpus callosum |
| Central corpus callosum | Corpus callosum |
| Middle anterior corpus callosum | Corpus callosum |
| Anterior corpus callosum | Corpus callosum |
| Pallidum | Sub-cortical |
| Hippocampus | Sub-cortical |
| Amygdala | Sub-cortical |
| Thalamus | Sub-cortical |
| Caudate | Sub-cortical |
| Accumbens | Sub-cortical |
| Ventral diencephalon | Sub-cortical |
| Banks of superior temporal sulcus | Temporal |
| Caudal anterior cingulate | Cingulate |
| Caudal middle frontal | Frontal |
| Cuneus | Occipital |
| Entorhinal | Temporal |
| Fusiform | Temporal |
| Inferior parietal | Parietal |
| Inferior temporal | Temporal |
| Isthmus | Parietal |
| Lateral occipital | Occipital |
| Lateral orbitofrontal | Frontal |
| Lingual | Occipital |
| Medial orbitofrontal | Frontal |
| Middle temporal | Temporal |
| Parahippocampal | Temporal |
| Paracentral | Frontal |
| Pars opercularis | Frontal |
| Pars orbitalis | Frontal |
| Pars triangularis | Frontal |
| Pericalcarine | Occipital |
| Postcentral | Parietal |
| Posterior cingulate | Cingulate |
| Precentral | Frontal |
| Precuneus | Parietal |
| Rostral anterior cingulate | Cingulate |
| Rostral middle frontal | Frontal |
| Superior frontal | Frontal |
| Superior parietal | Parietal |
| Superior temporal | Temporal |
| Supramarginal | Parietal |
| Frontal pole | Frontal |
| Temporal pole | Temporal |
| Transverse temporal | Temporal |
| Insula | - |

The Desikan-Killiany atlas of FreeSurfer V 5.3 was used to parcellate discrete anatomical regions of interest (ROIs)
